# Divergent coupling mechanism of precipitation on plant community multifunction across alpine grassland on the Tibetan Plateau

**DOI:** 10.3389/fpls.2022.1122140

**Published:** 2023-01-20

**Authors:** Miao Liu, Yang Li, Le Sun, Ziyin Du, Wencheng Li, Lin Zhang, Jinniu Wang, Ji Chen

**Affiliations:** ^1^ College of Grassland Science and Technology, China Agricultural University, Beijing, China; ^2^ State Key Laboratory of Tibetan Plateau Earth System, Environment and Resources (TPESER), Institute of Tibetan Plateau Research, Chinese Academy of Sciences, Beijing, China; ^3^ School of Geographical Sciences, China West Normal University, Nanchong, China; ^4^ College of Resources and Environment, Tibet Agriculture and Animal Husbandry University, Nyingchi, China; ^5^ Chengdu Institute of Biology, Chinese Academy of Sciences, Chengdu, China; ^6^ Department of Agroecology, Aarhus University, Tjele, Denmark; ^7^ Aarhus University Centre for Circular Bioeconomy, Aarhus University, Tjele, Denmark; ^8^ Climate Interdisciplinary Centre for Climate Change, Aarhus University, Roskilde, Denmark

**Keywords:** alpine meadow, alpine steppe, community-weighted mean trait, ecological stoichiometry, leaf functional traits, transect

## Abstract

**Introduction:**

It is essential to understand plant adaptive strategies on plant stoichiometric traits at the species level rather than at the community level under various environmental conditions across the Tibetan Plateau (TP).

**Methods:**

Here, plant community function and edaphic and meteorological factors were collected at 111 sites along an extensive water–heat gradient during the peak growing season in 2015. Community-weighted mean trait (CWM) was introduced to illuminating dynamics of the functional trait at the community level.

**Results:**

Our results indicated that plant functional traits, including CWM-leaf total carbon (CWM_LTC), CWM-leaf total nitrogen (CWM_LTN), and CWM-leaf total phosphorus (CWM_LTP), showed similar and comparatively marked increases from alpine meadow (AM) to alpine steppe (AS). Moreover, since the tightly coordinated variation among each plant functional trait of AM was higher than that of AS, a more stable coupling mechanism of these plant functional traits could be observed in AM under a long-term evolutionary habit. Specifically, there was higher annual mean precipitation (AMP) in AM than that in AS significantly (*P* < 0.01), and AMP was significantly correlated with soil moisture and soil total phosphorus in AM. Generally, our findings suggest that precipitation determines divergent coupling plant community function in both AS and AM.

## 1 Introduction

Ecosystem processes, material cycling, and energy flow relied on the species and functional composition of ecosystems, especially in certain plant communities (Hooper et al., 2005; Suding et al., 2008). Plant functional traits were commonly utilized to evaluate the ecosystem health, resilience, and other important facets of ecosystem functions (Sun et al., 2022). Essentially, the plant functional traits were generally considered to be a species adaptive outcome within a differentiated environment. And the range of functional trait values was shaped by these adaptive processes through habitat filtering or interspecific competition for available resources within communities (Faucon et al., 2017). Specifically, it not only reflects the survival strategies of plants in response to changes in their habitats to explore the linkage between plant functional traits and environmental variables but also enables accurate estimation of processes and functions in multiple ecosystems (Lavorel et al., 2007; Funk et al., 2017). This issue has become one of the most popular ecological research hot spots at different spatial and temporal scales (Díaz and Cabido, 2001; de Bello et al., 2010; Bjorkman et al., 2018), for it offers precise measurements of many ecosystem processes and responses directly or indirectly revealing from individual to ecosystem-level functions (Kremen, 2005; Suding et al., 2008). Plant functional traits have been proposed as a direct way of predicting changes in ecosystem processes through the changes in plant communities in responding to global change factors (Fortunel et al., 2009). In the previous studies, they have suggested that plant functions can impart ecosystem resistance and resilience to climate extremes, especially when the number of species with traits associated with escape strategies is high (McDowell et al., 2008; Griffin-Nolan et al., 2019). Thus, it is essential to conduct consistent and accurate measurements for exploring the linkage between plant functional traits and ecosystem functions, especially with the growing threat of climate change and human disturbance (Imbert et al., 2020; Schlickmann et al., 2020). Previous research has demonstrated that the community-weighted mean trait (CWM) could reflect the anticipant trait value of a randomly sampled individual from a community (Garnier et al., 2004), and it described the conditions of plant community function, which can not only the track plant adaptive strategy to the changing environment but also play important roles in shaping ecosystem functions (Liu et al., 2020). Therefore, it will be a more comprehensive way to study the coupling patterns and driven mechanisms of community plant function along natural water and heat gradients by CWM.

Ecological stoichiometry, as a set of important plant functional traits, could be used to characterize coupling relationships of plant community functions. As we know that the complicated interactions among C, N, and P were performed in terrestrial ecosystems, plant growth was basically a process of maintaining its own stability by accumulating elements (mainly C, N, and P) and adjusting their relative proportions (Dai et al., 2022). Furthermore, green leaves were responsible for the functioning of terrestrial plants from individual to community, and leaf N, P, and N:P ratio were core leaf traits that played critical roles in plant survival, growth, and development (Wright et al., 2004; Wright et al., 2005). Currently, terrestrial C and N coupling has received gradually increased attention due to intensifying N limitation on future carbon sequestration in terrestrial ecosystems (Reich et al., 2006). Apparently, the balance among C, N, and P exerted a profound impact on diverse plant functions, which was able to quickly respond to the dynamics of nutrient supply *via* its physiological adaptability. Previous studies have explored the coupling patterns of plant N and P (Zhou et al., 2020), leaf N and P (Li et al., 2022), and root N and P in alpine grassland (Ma et al., 2020). And they have illustrated that water and heat play a crucial effect in determining the coupling and decoupling process of C, N, and P biogeochemical cycles. As mentioned above, there is still a lack of a comprehensive way to study the coupling patterns and driven mechanisms of community plant function, in which CWM might be a viable pathway.

The Tibetan Plateau (TP) has a wide water and heat gradient from east to west, making it an ideal region to explore the coupling patterns and underlying mechanisms of plant community function across alpine grassland. Additionally, CWM was introduced to better explain the functional trait variations at the community level. Hence, we hypothesized that precipitation determines the coupling mechanism of the plant community function across alpine grassland in the TP. A transect survey was conducted along water and heat gradients across the TP. Moreover, plant C, N, and P in communities, together with edaphic and meteorological factors, were measured to test our assumption. The objectives of this study are as follows: 1) manifest the coupling patterns of plant functional traits across the whole transect region and 2) reveal the internal mechanisms among meteorological factors, soil resources, and plant community function of the proven coupling performance over the TP.

## 2 Materials and methods

### 2.1 Study area

As the third pole of the world, the TP is a sensitive and vulnerable ecosystem to global climate change with a wide altitude of 3,030–5,000 m (Zhou et al., 2021). The area of the TP is about 2.5 million km^2^, covering Tibet, Qinghai, Sichuan, and Gansu province (79°58′-102°46′E, 28°55′-37°19′N) (Sun et al., 2018). Due to the complex geographical and geomorphological environment, the precipitation and temperature of this region are characterized by dynamic gradients with a mean annual precipitation of 114–992 mm and mean annual temperature of -4.41°C to 6.33°C (Sun et al., 2020). Furthermore, precipitation is mainly concentrated in the growing season from May to September, and the average monthly temperature varies significantly, with the coldest and warmest month occurring in January and July, respectively (Zhou et al., 2021). According to the grassland classification in China, the TP has almost all grassland types, two of which are alpine meadow (AM) and alpine steppe (AS) as the hot spots concerned by government and researchers (Sun et al., 2019). The AM in the research area is dominated by *Kobresia tibetica*, *Stipa capillata*, and *Carex lasiocarpa*, while the AS is dominated by *Stipa purpurea*, *Stipa subsessiliflora*, and *Carex sutschanensis*. The soil type is mainly classified as alpine meadow soil in AM and subalpine steppe soil in AS.

### 2.2 Sample collection and analysis

**Figure 1:**
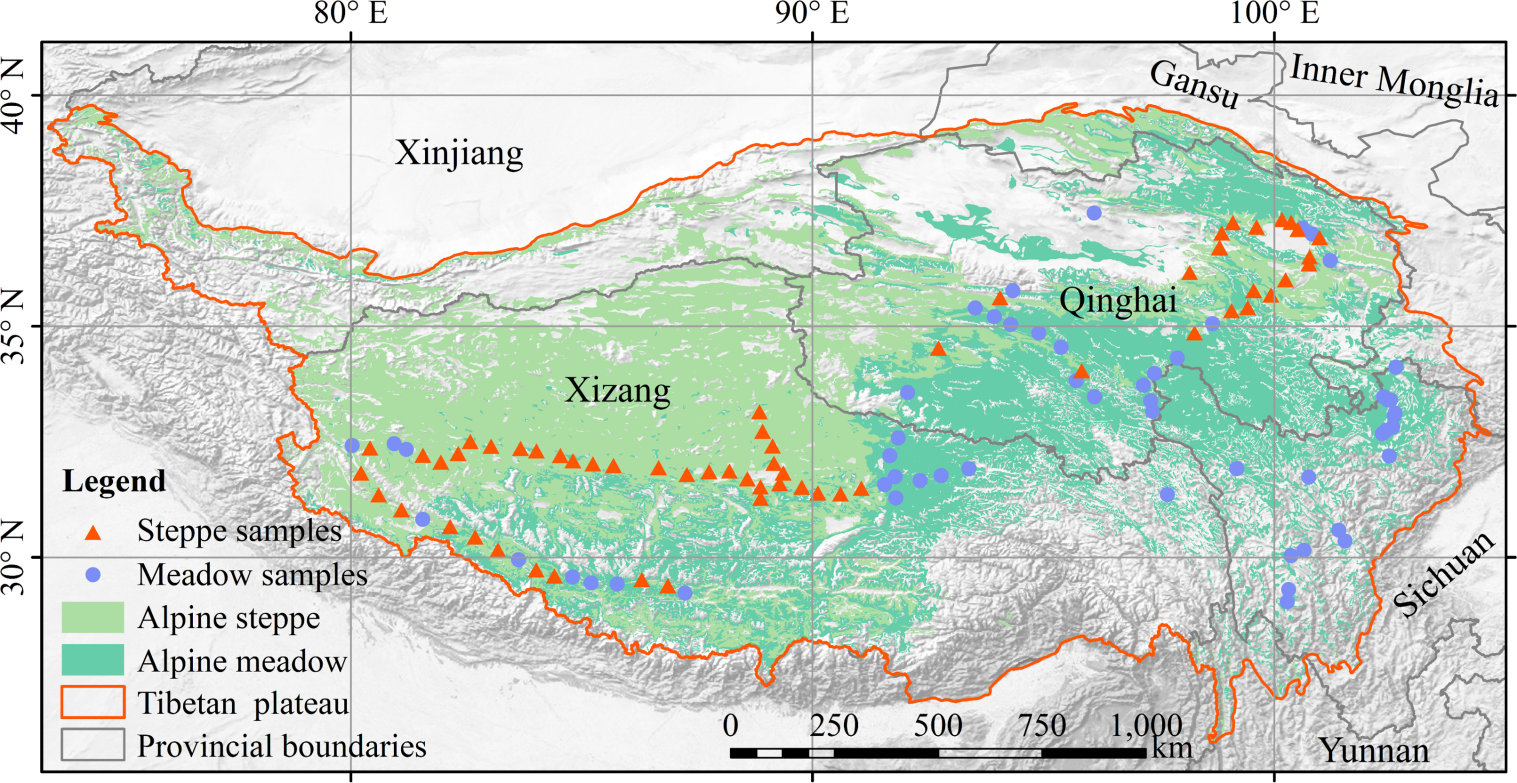
Locations and landscape of the sampling sites across the Tibetan Plateau.

From July to mid-August 2015, an approximately 5,000-km-long transect survey was conducted during the plant growing season across the whole natural alpine grasslands in the TP. A total of 111 sites were investigated along the transect covering two grassland types, including AM and AS (**Figure 1**). At each site (10 m × 10 m), three plots (0.5 m × 0.5 m) were randomly sorted with similar topography. The species information was identified for all species, and the vegetation coverage, vegetation density, and plant height were measured within each plot. The plant samples were harvested by clipping the aboveground part according to different species, then sun-drying was applied in the field and oven-drying was performed at 65°C in the laboratory until a constant weight was achieved. After grounding the dried plant samples by a ball mill (NM200; Retsch, Haan, Germany), the leaf total carbon (LTC) and leaf total nitrogen (LTN) were measured using a Vario MACRO cube elemental analyzer (Elementar Analysensysteme GmbH, Germany), and leaf total phosphorus (LTP) was measured using phosphor molybdate blue spectrophotometry (He et al., 2008). Moreover, soil samples were collected using a soil auger to determine soil properties at the 30-cm soil depth. After being air-dried and sieved using 2-mm mesh, the soil samples were carefully handpicked to extract the surface organic materials and fine roots for the analysis of soil physical and chemical properties. Then, each mixed soil sample was divided into two parts: one part was oven-dried at 105°C to a constant weight to test the gravimetric soil moisture (SM) and soil bulk density (SBD); the other was grounded in a ball mill for the analysis of soil total organic carbon (SOC), soil total nitrogen (STN), soil-available nitrogen (SAN), soil total phosphorus (STP), and soil-available phosphorus (SAP). Soil properties were measured according to all standard protocols (Bao, 2000).

### 2.3 Meteorological data

Meteorological factors of annual mean temperature (AMT) and annual mean precipitation (AMP) were spatially interpolated by the Anusplin 4.2 (Centre for Resource and Environmental Studies, Australian National University, Canberra) with a spatial resolution of 1 km. The longitude and latitude for the sampling sites were acquired *via* the Global Positioning System (GPS). Then, the AMT and AMP of the sampled sites were extracted *via* ArcGIS 10.2 from the spatially interpolated meteorological product based on the geographic information.

### 2.4 Data analysis

In order to determine the coupling mechanisms of the plant community function, we firstly quantified the functional diversity at the plant community level using community-weighted mean (CWM values). The CWM for each of the three plant functional traits was calculated as follows:


$$CWM=\sum_{i=1}^{s}P_{i}\times trait_{i}$$

where *P_i_* is the relative abundance of species i (i = 1, 2, …, S) in the community, and *trait_i_* is the trait value for species i (Lavorel et al., 2008). From an ecological viewpoint, this metric is strongly driven by the trait values of the dominant species within a community and is directly related to the mass ratio hypothesis (Grime, 1998). The traits of dominant species, which largely determined the ecosystem processes, could be indicated by a significant effect of CWM on ecosystem functions (Mokany et al., 2008).

Secondly, both normality test and the one-way ANOVA were performed using SPSS 19.0 software (SPSS Inc., Chicago, IL, USA) to detect the data distribution and differences in plant functional traits, including the community-weighted mean of leaf total carbon (CWM_LTC), community-weighted mean of leaf total nitrogen (CWM_LTN), and community-weighted mean of leaf total phosphorus (CWM_LTP) between AM and AS. Moreover, we explored the relationships between CWM_LTC, CWM_LTN, and CWM_LTP. The linear regression analysis was used to explain the linkages among them using R software (R Core Team, 2021) with the “*ggplot 2*” package.

Finally, the linear regression analysis was performed using R to reveal the relationships of the plant functional traits with AMP in different patterns. The “*pheatmap*” package in R software was utilized for exploring the correlations between CWM_LTC, CWM_LTN, CWM_LTP, and soil properties. Then, we screened the critical soil properties that were significantly associated with the coupling of plant functional traits through matrix correlation and revealed their relationships with precipitation. A map of the TP was plotted in ArcGIS 10.2, and statistical analysis was completed using SPSS 19.0 software (SPSS Inc., Chicago, IL, USA).

## 3 Results

### 3.1 Plant functional trait in alpine meadow and alpine steppe

**Figure 2:**
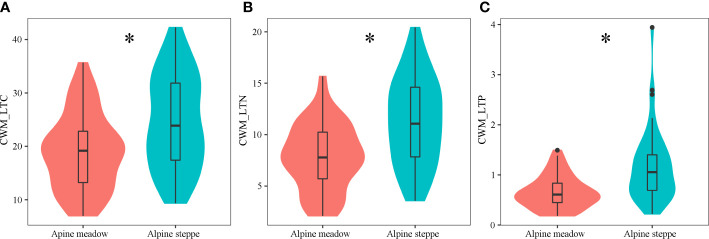
Shifts of plant functional traits in different grassland types. Panels **(A–C)** represent CWM_LTC, CWM_LTN, and CWM_LTP, respectively. The symbol * denotes the significant differences between different grassland types (*P*< 0.05). CWM_LTC, CWM_LTN, and CWM_LTP represent community-weighted mean of leaf total carbon, community-weighted mean of leaf total nitrogen, and community-weighted mean of leaf total phosphorus, respectively.

The mean value of CWM_LTC, CWM_LTN, and CWM_LTP across AM was 18.87, 7.81, and 0.66 while those across AS was 24.53, 11.28, and 1.13, respectively (**Figure 2**). As expected, all of the CWM_LTC, CWM_LTN, and CWM_LTP presented significant differences between AM and AS (*P*< 0.05). These functional traits showed similar and comparatively marked increases from AM to AS.

### 3.2 Coupling relationship among plant functional traits across alpine grasslands

**Figure 3:**
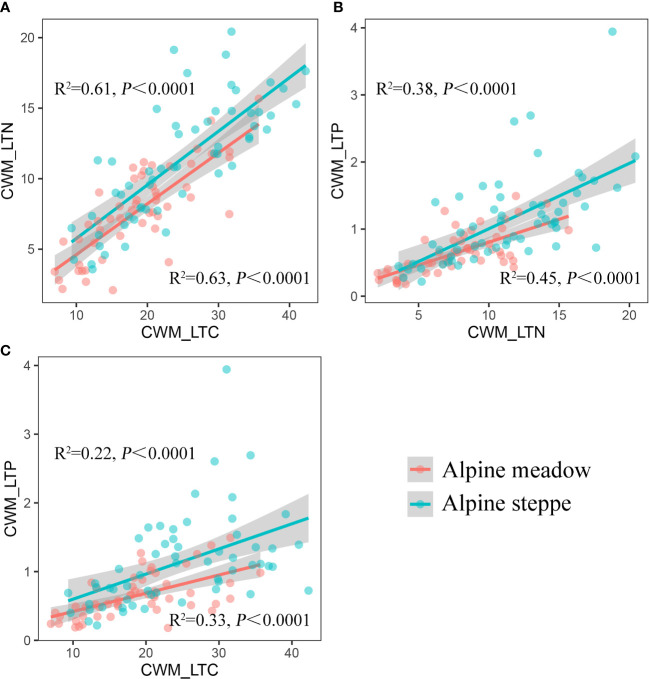
The linear fits of **(A)** CWM_LTN to CWM_LTC, **(B)** CWM_LTP to CWM_LTN, and **(C)** CWM_LTP to CWM_LTC. CWM_LTC, CWM_LTN, and CWM_LTP represent community-weighted mean of leaf total carbon, community-weighted mean of leaf total nitrogen, and community-weighted mean of leaf total phosphorus, respectively.

The coupling degree among each plant functional trait of AM was higher than that of AS, which could be reflected by the R^2^ value. The highest R^2^ values were found in the relationships between CWM_LTC and CWM_LTN in both vegetation types (**Figure 3A**). The most predominant differences occurred in the coupling relationships between CWM_LTC and CWM_LTP (R^2^ = 0.33 in AM vs. R^2^ = 0.22 in AS, **Figure 3B**) and between CWM_LTN and CWM_LTP (R^2^ = 0.45 in AM vs. R^2^ = 0.38 in AS, **Figure 3C**).

### 3.3 Links of plant stoichiometry traits to annual mean precipitation and annual mean temperature

**Figure 4:**
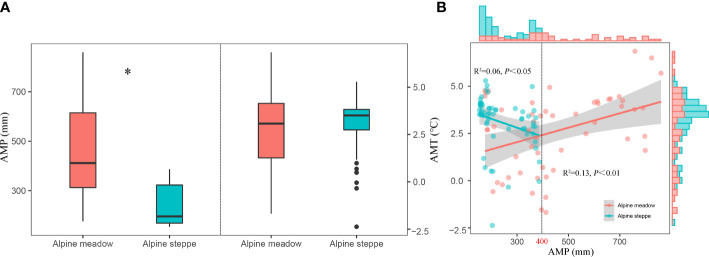
The differences of AMP and AMT **(A)** and their correlations in alpine steppe and alpine meadow **(B)**. AMP and AMT represent annual mean precipitation and annual mean temperature, respectively. * represents P< 0.05.

AMP showed a declining trend from AM to AS with its mean value of 457.81 mm and 238.20 mm, respectively, while AMT did not vary between different vegetation types (**Figure 4A**). Moreover, the relationships of AMP and AMT differ between vegetation types, i.e., a significant negative relation was detected in AS (*P*< 0.05), and an opposite phenomenon was found in AM (*P*< 0.01) (**Figure 4B**).

**Figure 5:**
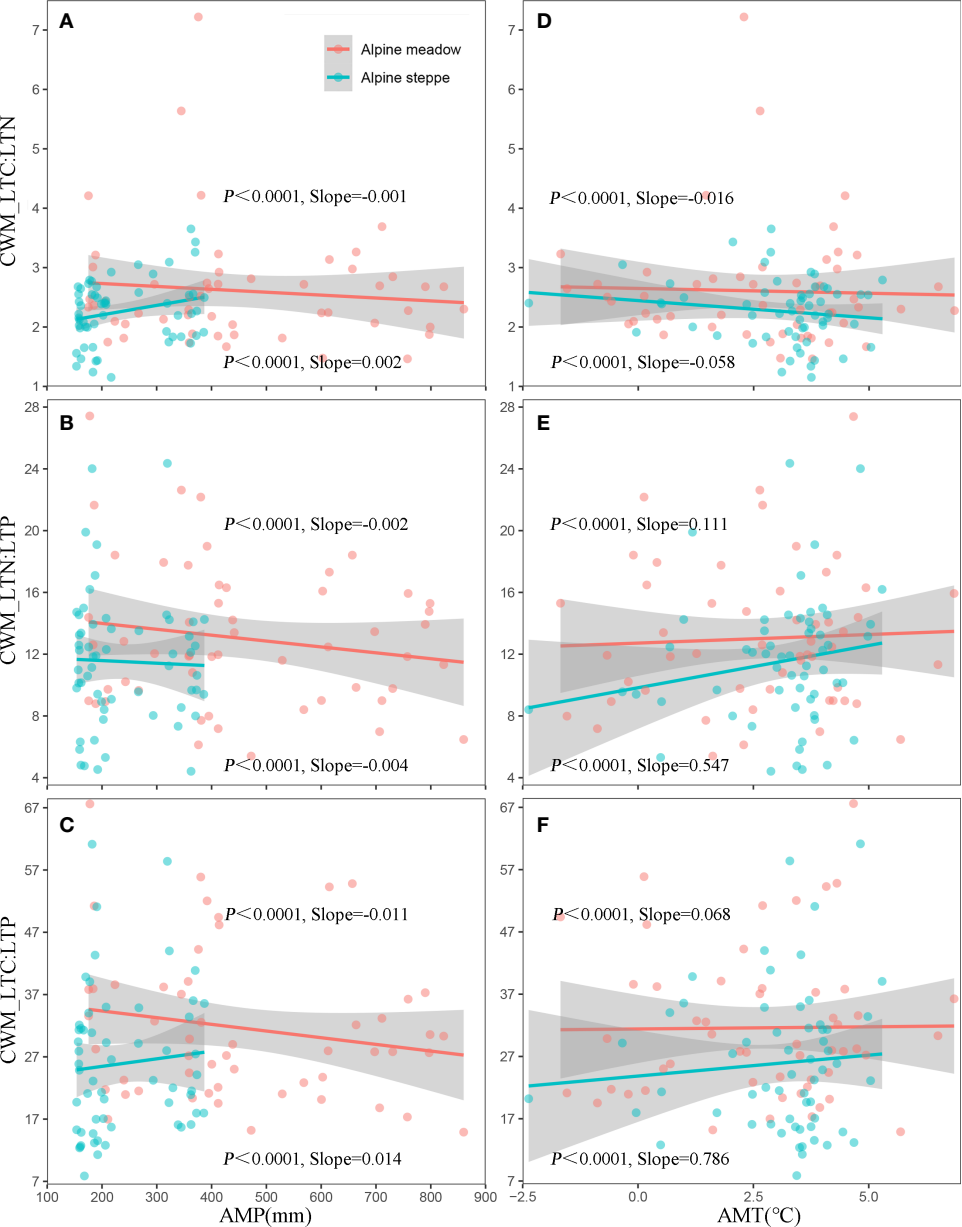
The linear fits of AMP to **(A)** CWM_LTC, **(B)** CWM_LTN, and **(C)** CWM_LTC and AMT to **(D)** CWM_LTC, **(E)** CWM_LTN, and **(F)** CWM_LTP. CWM_LTC, CWM_LTN, and CWM_LTP represent community-weighted mean of leaf total carbon, community-weighted mean of leaf total nitrogen, and community-weighted mean of leaf total phosphorus, respectively.

Additionally, the dynamics of CWM_LTC : LTN, CWM_LTN : LTP, and CWM_LTC : LTP along AMP and AMT were analyzed in AM and AS. Specifically, the slopes of CWM_LTC : LTN, CWM_LTN : LTP, and CWM_LTC : LTP along AMT were significantly greater than those along AMP (*P*< 0.0001). That is, the change rates of all plant stoichiometries varied little with AMP, while these change rates were comparatively large with AMT. Overall, the slopes in AM were significantly lower than those in AS (**Figure 5**), indicating a more stable coupling mechanism of these plant functional traits in AM under a long-term evolutionary habitat.

### 3.4 Soil properties affect plant stoichiometry traits in alpine grasslands

All plant stoichiometry traits and soil properties in AM were significantly different from those in AS except for CWM_LTN : LTP and SAP. The mean values of plant stoichiometry traits and soil properties in AM were all greater than those in AS except SBD (**Table 1**).

**Table 1:** Differences of plant stoichiometry traits and soil properties in the two different alpine grassland types.

	AM	AS		AM	AS
CWM_LTC : LTN	2.61 ± 0.98^a^	2.27 ± 0.54^b^	SOC (g/kg)	36.93 ± 31.29^a^	13.79 ± 12.20^b^
CWM_LTN : LTP	13.01 ± 4.64^a^	11.52 ± 4.25^a^	STN (g/kg)	2.31 ± 1.60^a^	1.10 ± 0.93^b^
CWM_LTC : LTP	33.30 ± 17.78^a^	26.04 ± 11.75^b^	STP (g/kg)	0.62 ± 0.37^a^	0.36 ± 0.21^b^
SM (%)	27.73 ± 25.42^a^	11.37 ± 6.11^b^	SAN (mg/kg)	198.22 ± 131.15^a^	73.86 ± 65.01^b^
SBD (g/cm^3^)	0.98 ± 0.29^a^	1.32 ± 0.23^b^	SAP (mg/kg)	2.79 ± 1.74^a^	2.28 ± 1.59^a^

AM, alpine meadow; AS, alpine steppe; CWM_LTC, community-weighted mean of leaf total carbon; CWM_LTN, community-weighted mean of leaf total nitrogen; CWM_LTP, community-weighted mean of leaf total phosphorus; SM, soil moisture; SBD, soil bulk density; SOC, soil total organic carbon; STN, soil total nitrogen; STP, soil total phosphorus; SAN, soil-available nitrogen; SAP, and soil-available phosphorus. Different letters indicate significant differences at the 0.05 level between different grassland types.

**Figure 6:**
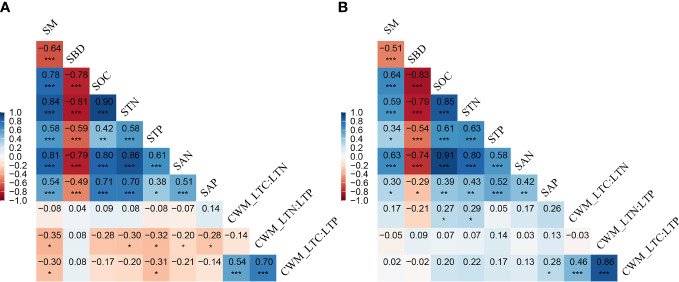
Correlation between plant stoichiometry traits and soil properties. Plant stoichiometry traits include CWM_LTC : LTN, CWM_LTN : LTP, and CWM_LTC : LTP, and soil properties include soil moisture (SM), soil bulk density (SBD), soil total organic carbon (SOC), total soil nitrogen (STN), total soil phosphorus (STP), soil-available nitrogen (SAN), and soil-available phosphorus (SAP). * represents *P*< 0.05, ** represents *P*< 0.01, *** represents *P*< 0.001, **(A)** alpine meadow, **(B)** alpine steppe.

CWM_LTC : LTN was not related to any of the soil properties in AM (**Figure 6A**) but showed positive correlations with SOC and STN in AS (**Figure 6B**). CWM_LTN : LTP exhibited negative relationships to soil properties of SM, STN, STP, and SAP in AM; however, all of these significant correlations disappeared in AS. CWM_LTC : LTP showed negative correlations with SM and STP, while it was positively correlated with SAP.

### 3.5 The relationships of AMP to soil properties

**Figure 7:**
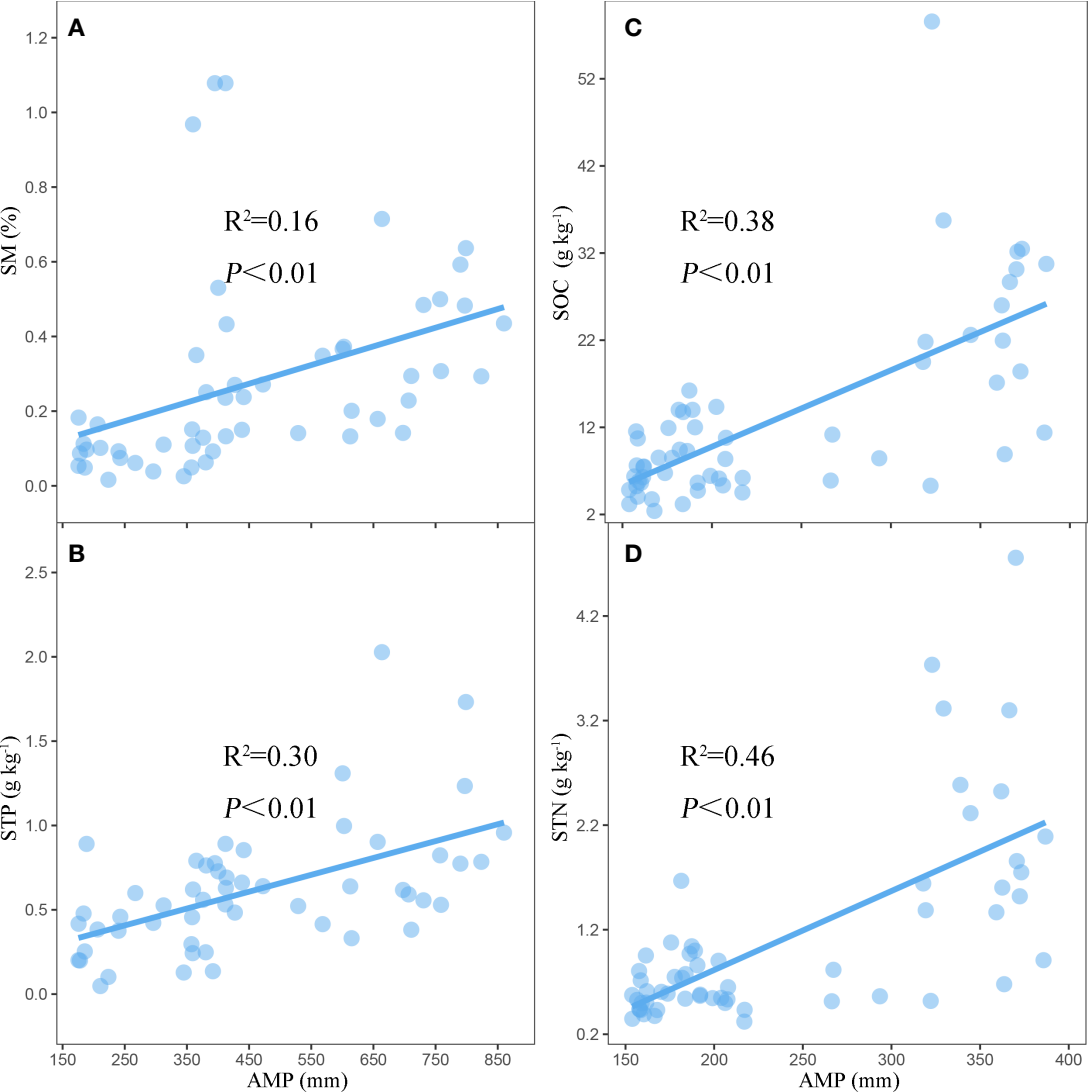
The linear fits of AMP to **(A)** SM and **(B)** STP in AM and SOC **(C)** and STN **(D)** in AS. SM, soil moisture; STP, soil total phosphorus; SOC, soil organic carbon; STN, soil total nitrogen.

Linear regression analysis revealed that SM (R^2^ = 0.16, P < 0.01, **Figure 7A**) and STP (R^2^ = 0.30, P < 0.01, **Figure 7B**) were positively correlated with AMP in AM. However, in the AS, the SOC and STN showed significant and positive relationships with AMP (R^2^ = 0.38, P < 0.01, **Figure 7C**; R^2^ = 0.46, P < 0.01, **Figure 7D**).

## 4 Discussion

Plant community functional composition responds to environmental variation and influences ecosystem processes, such as nutrient cycling and decomposition (Díaz and Cabido, 2001; Griffin-Nolan et al., 2019). We explored the effects of climate and soil factors on the coupling mechanism of plant community functions in two different alpine grassland types. The main findings indicated that the precipitation documented the coupling mechanism of plant functional traits in alpine grassland. For soil properties, SM and STP were the key factors controlling the coupling mechanism of plant functional traits in AM, while SOC and STN were the main regulating factors in AS.

### 4.1 The divergent coupling mechanism of plant functional traits

C, N, and P cycling constrains most ecosystem processes (Aerts and Chapin, 1999). Strong positive correlations between N and P were found in many previous studies, while no correlations were detected between C and N and between C and P (Thompson et al., 1997; Reich and Oleksyn, 2004; Han et al., 2005; Zheng and Shangguan, 2007). Nevertheless, the relationships for the plant functional traits are quite different from those for nutrient cycling. We found significantly positive correlations between CWM_LTC and CWM_LTN, CWM_LTN and CWM_LTP, and CWM_LTC and CWM_LTP in both AM and AS (**Figures 3A–C**). It demonstrated that leaf N and P are usually interrelated and highly positively correlated with each other (Güsewell, 2004; He et al., 2008). The coupling of leaf N and P was mainly mediated through the whole plant signaling mechanism induced by the adjustment of N and P uptake rates, which has both positive and negative effects: the rate of N uptake increases in N-deficient plants, while it is opposite in P-deficient plants (Imsande and Touraine, 1994; Aerts and Chapin, 1999). Moreover, the supply shortage in P tends to limit the N fixation and C storage in the long term (Vitousek and Howarth, 1991; Tiessen, 2008; Wang et al., 2010), suggesting a high positive correlation between P and N and P and C. Our results confirmed previous studies that there was an indirect effect of C economy on the linkage between N and P (Eckstein and Karlsson, 2001).

### 4.2 Precipitation dominates the coupling plant functional traits in alpine grasslands

Anthropogenic climate change-induced changes in precipitation patterns have universal effects on plant community functions and processes in terrestrial ecosystems (Huxman et al., 2004; Bahn et al., 2014). Meanwhile, spatial variability of precipitation has a critical role in determining plant community structures in grassland ecosystems (Pierce et al., 2019). The climate of the TP has substantially changed in recent decades. On the one hand, the average annual temperature has risen by 0.4°C per decade over the past 50 years over twice the rate of global temperature rise. On the other hand, interannual variability in precipitation is increasingly significant on the TP (Shen et al., 2015). Previous studies have suggested that variations in precipitation patterns can lead to changes in traits and species richness, resulting in a gradient in plant distribution or compositions along the precipitation (Griffin-Nolan et al., 2018). Precipitation is linearly characterized along a natural gradient, and some plant functional traits such as leaf traits, as predictors of plant community, closely respond to large-scale precipitation changes at the community level (Sandel et al., 2010; Le Bagousse-Pinguet et al., 2017). Our study was carried out on the TP where precipitation plays an important role in determining the coupling degree of CWM_LTC, CWM_LTN, and CWM_LTP across the alpine grassland (**Figure 5**). Essentially, precipitation variabilities and differences determine the competing interactions between the patterns of plant community functional traits and diversity assemblages in AM and AS (Zuo et al., 2021). Specifically, the coupling level among each plant functional trait of AM was higher than that of AS (**Figures 5A–C**), and it was probably due to the relatively better hydrothermal conditions in AM (Zhou et al., 2020). It is the precipitation, the major limiting factor for plant growth over the TP, that promotes plant photosynthesis and nutrient transportation and exerts large impacts on plant stoichiometry traits through mediating soil moisture for grasslands (Sun et al., 2013). In arid environments, the reduced rainfall resulting in low soil water availability and nutrient mobility was responsible for the high variability of plant stoichiometry traits and then causing the decoupling of plant community function in AS (Güsewell, 2004; Elser et al., 2007). Thereby, more stable coupling patterns of these plant functional traits were detected in AM under a long-term evolutionary habitat.

Although there were correlations between coupling of plant functional traits and climate factors, the linear fitting indicated that the slopes of CWM_LTC : LTN, CWM_LTN : LTP, and CWM_LTC : LTP along AMT were significantly greater than those along AMP (*P*< 0.0001) (**Figure 5**). The above results indicated a more stable coupling mechanism of plant functional traits in AMP. In other words, precipitation was the key climatic factor determining the coupling degree of plant functional traits in alpine grasslands. Our results were similar to previous findings that the coupling among biogeochemical cycles in drylands would become particularly vulnerable in the face of rapid climate change (Delgado et al., 2013). Meanwhile, the magnitude of CWM_LTC, CWM_LTN, and CWM_LTP and their ratios reflected the adaptability of plants to local environmental conditions. It was a reasonable assumption that precipitation acts as a prior limiting factor for plant growth and might bring a greater effect on leaf element concentrations compared to temperature in alpine grasslands (Fan et al., 2016). Furthermore, we suggested that AMP might not affect the degree of coupling directly but rather affected the functional plant traits *via* influencing the soil nutrient status. Our results were consistent with those of previous studies, which demonstrated that precipitation affected plant functional traits mainly through affecting the soil nutrients (He et al., 2008).

### 4.3 Soil physicochemical properties mediate plant functional traits

Plant functional traits were affected by soil physical and chemical properties to some extent (Guo et al., 2022). Soil nutrients directly affected the physiological activity and species composition of the plant community and regulated the ecosystem structures and functions. In particular, leaf C, N, and P content not only reflected the soil nutrient availability (Asplund and Wardle, 2014) but also was impacted by soil N and P supply (Han et al., 2011; Chen et al., 2013) because plants mainly obtained these elements (especially N and P) from soil. The distinct geothermal gradients and hydrological cycles on the TP could exert significant effects on biogeochemical processes in regulating soil C, N, and P cycles, which might differ from other regions. Moreover, the coupling of plant functional traits was an important index in the description of community vegetation structure, functions, and nutrient limitation (Koerselman and Meuleman, 1996; Thompson et al., 1997; Güsewell, 2004; Reich and Oleksyn, 2004). Our study showed that the plant functional traits of CWM_LTC : LTN, CWM_LTN : LTP, and CWM_LTC : LTP in AS were lower than those in AM (**Table 1**). One possible reason should be the low N content and effectiveness of AS soil, as well as the low C content in the returned nutrients from litter (Pan et al., 2015). Meanwhile, the lower CWM_LTN and CWM_LTP in AM than those in AS (**Figures 2B, C** **)** agree with their fast growth, since the relatively low nutrient levels could satisfy the rapid plant growth in the suitable hydrothermal conditions in AM (Güsewell, 2004). Additionally, the leaf N:P ratio of the plant community was assumed to reflect the soil N status. However, no significant positive correlation was found between the soil total N and N:P ratio of leaves in AS (Zhang et al., 2011).

Generally, studies on the relationships between plant functional traits and soil properties are critical for understanding the common characteristics in the plant community that respond to global changes. In this study, we found that the coupling of plant functional traits, including CWM_LTC : LTN, CWM_LTN : LTP, and CWM_LTC : LTP, was closely related to soil physicochemical properties across the alpine grasslands. This was consistent with previous findings by Rastetter et al. (1991) and Novotny et al. (2007), who found that the changes in soil nutrients would lead to shifts in plant C, N, and P and their ratios. The leaf economics spectrum revealed that an increased availability of resources related to soil fertility will select for similar leaf trait syndromes (Wright et al., 2004; Díaz et al., 2016; Both et al., 2019). Interestingly, the soil factors, such as SM and STP, are key drivers of the coupling of plant functional traits in AM (**Figure 7A**), while it turned to SOC and STN in AS (**Figure 7B**). On the one hand, researchers reported that a higher soil water content may limit the microbial decomposition of soil organic matter in AM, so that soil organic matter is better preserved compared to AS (Geng et al., 2017). On the other hand, the precipitation is much heavier in AM than that in AS, which may enhance the SOC and STN by the increased plant productivity and substrate effectiveness (Tian et al., 2017). Furthermore, our results suggested that the soil properties, including SM, STP, SOC, and STN, were positively correlated with precipitation across the alpine grasslands (**Figures 7A–D**). Precipitation was a direct source of soil moisture, which enhanced the microbial activities and thus increased the nutrient availability to plants by increasing water availability (Allison and Treseder, 2008). These results were consistent with our hypothesis, i.e., precipitation determines the coupling mechanism of plant community function by altering soil properties across alpine grassland in the TP. Therefore, it is of great significance to further investigate the drivers of the coupling of plant functional traits under different scenarios of future precipitation changes.

## 5 Conclusion

Our results indicated that AM possessed a more robust coupling mechanism of plant functional traits under a long-term evolutionary habitat due to the relatively favorable hydrothermal conditions. Consequently, we concluded that precipitation dominates coupling mechanisms of plant community function through affecting soil conditions across alpine grassland in the TP. Therefore, we suggested that the integrated effects of precipitation and temperature instead of AMP or AMT individually should be taken into account when investigating the underlying mechanism of plant community functions across alpine grasslands in the TP.

## Data availability statement

The original contributions presented in the study are included in the article/supplementary material. Further inquiries can be directed to the corresponding author.

## Author contributions

ML contributed to the study design. ML, YL, LS, ZD, WL, LZ. JW and JC were involved in drafting the manuscript and agree to be accountable for the work. All authors contributed to the article and approved the submitted version.
